# Supplementary material for: Toxicity of UV Filter Benzophenone-3 in Brine Shrimp Nauplii (Artemia salina) and Zebrafish (Danio rerio) Embryos
Source: J Xenobiot. 2024 Apr 29;14(2):537–53. doi: 10.3390/jox14020032 (PMC11130858; doi:10.3390/jox14020032)
Supplement: Supplementary file 1 [file jox-14-00032-s001.zip › jox-2866738-supplementary.pdf]

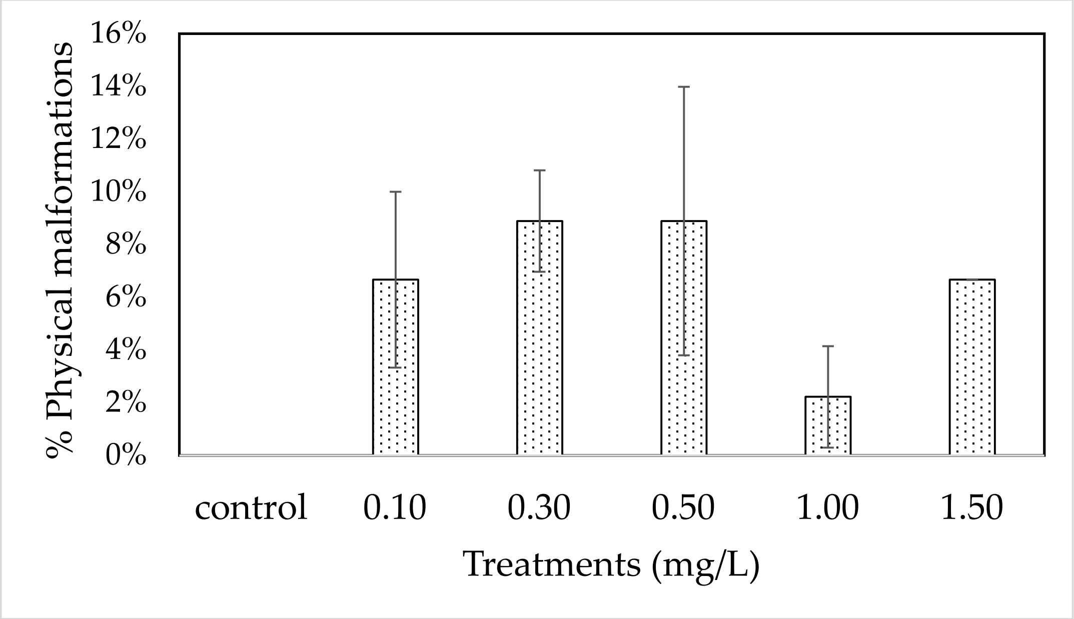

Figure. S1. Percentage of physical malformations in zebrafish larvae exposed to BP-3.

The groups that presented a higher percentage of physical malformations were at concentrations of 0.30 and 0.50 mg/L, with 9% malformation. No deformations were observed in the control groups. All groups exposed to BP-3 presented some physical malformations. The group that presented the lowest percentage of physical malformation was the 1.0 mg/L group, with 2%. Significant differences were found with a value of p=0.008. n=3
